# StandFood: Standardization of Foods Using a Semi-Automatic System for Classifying and Describing Foods According to FoodEx2

**DOI:** 10.3390/nu9060542

**Published:** 2017-05-26

**Authors:** Tome Eftimov, Peter Korošec, Barbara Koroušić Seljak

**Affiliations:** 1 Computer Systems Department, Jožef Stefan Institute, Jamova cesta 39, 1000 Ljubljana, Slovenia; peter.korosec@ijs.si (P.K.); barbara.korousic@ijs.si (B.K.S.); 2 Jožef Stefan International Postgraduate School, Jamova cesta 39, 1000 Ljubljana, Slovenia; 3 Faculty of Mathematics, Natural Science and Information Technologies, Glagoljaška ulica 8, 6000 Koper, Slovenia

**Keywords:** food standardization, food classification, food description, FoodEx2, machine learning, natural language processing

## Abstract

The European Food Safety Authority has developed a standardized food classification and description system called FoodEx2. It uses facets to describe food properties and aspects from various perspectives, making it easier to compare food consumption data from different sources and perform more detailed data analyses. However, both food composition data and food consumption data, which need to be linked, are lacking in FoodEx2 because the process of classification and description has to be manually performed—a process that is laborious and requires good knowledge of the system and also good knowledge of food (composition, processing, marketing, etc.). In this paper, we introduce a semi-automatic system for classifying and describing foods according to FoodEx2, which consists of three parts. The first involves a machine learning approach and classifies foods into four FoodEx2 categories, with two for single foods: raw (r) and derivatives (d), and two for composite foods: simple (s) and aggregated (c). The second uses a natural language processing approach and probability theory to describe foods. The third combines the result from the first and the second part by defining post-processing rules in order to improve the result for the classification part. We tested the system using a set of food items (from Slovenia) manually-coded according to FoodEx2. The new semi-automatic system obtained an accuracy of 89% for the classification part and 79% for the description part, or an overall result of 79% for the whole system.

## 1. Introduction

In 2011, the European Food Safety Authority (EFSA) [1] introduced a comprehensive food classification and description system for exposure assessment, known as FoodEx1 [2], aimed at covering the need to describe food in data collections across different food safety domains. After a testing phase, in 2015, EFSA introduced a new version called FoodEx2 [2], in order to match the needs expressed by different users. The system consists of a lot of individual food items aggregated into food groups and broader food categories organized in a hierarchical relationship. In addition, it provides generic food descriptions that represent the minimum level of detail required for making intake or exposure assessments. The description is provided using facets, which are a collection of terms that describe the properties and aspects of foods from various perspectives.

FoodEx2 is applicable across different domains, in particular food consumption, chemical contaminants, pesticide residues, zoonoses, and food composition. It allows new forms of dietary assessment technology, and connects food intake and food composition data enabling the conversion of food intake into nutrient intake [3]. The system consists of three types of food categories representing three different levels in the food chain, which involve an increasing level of food processing, i.e., moving from raw commodities to derivatives to composite foods. Raw commodities (r) are the parts physically separated from a living source after harvesting (plants) or slaughtering (animals) and the processes it assumes to be involved, in the context of this classification, do not change the nature of food. Derivatives/ingredients (d) are food products obtained from raw commodities by applying a process that changes the nature of food. Composite foods are obtained by using more raw commodities and/or derivatives through processes that always involve recipes, simple composite food (s) and aggregated composite food (c). For example, simple composite foods include muesli, porridge, cereal bars, and jam, while examples of aggregated composite foods include pasta-based dishes, cakes, croissants, soups and salads. This means that processing has a critical role in defining whether a specific food item is raw, a derivative or a composite. Such processes are defined in the FoodEx2 technical report [2].

FoodEx2 includes two types of terms: list terms and facet descriptors. List terms are terms defining a food group, or, in a broader sense, taking account of the expanded scope of FoodEx2, a defined matrix for data reported in the food safety domain. List terms are the food groups appearing in the tree structure of the different hierarchies and may have different levels of aggregation. Depending on the scope of the groups, they may be reportable or not; in other words, some list terms are suitable for describing a food item, while coding data and other list terms are only suitable for browsing a hierarchy or creating a summary table for data analysis. The list terms that can be used to describe a food item are the base terms, because these can form the basis of a more detailed and complex FoodEx2 code for data reporting. List terms are represented by a code; for example, A0C75 is the code for “salmon”. Facet descriptors are elements of additional information included in or added to the list terms, each providing different options to describe a particular aspect of a food category, such as treatments received, production method, fat content, and qualitative information. FoodEx2 consists of 32 facets that can be either implicit or added. Implicit facets are applied to a specific food category, while added facets describe a characteristic of a specific food item. Each food category (r, d, s, or c) may have different facet descriptors. FoodEx2 code includes different pieces of coded information in a single string, which contains a code for a list term (mandatory), followed by a hashtag “#” and an unlimited sequence of facets, each separated by dollar characters “$”. For example, if the food item “nectar, orange” needs to be coded with FoodEx2, the code already existed in FoodEx2 and is A03BG. If additional information in the food description is presented, for example that this nectar is sugar free, organic, and fortified with calcium, the FoodEx2 code looks like this: A03BG#F09.A0EXH$F10.A077L$F21.A07SE. This code contains the information that this is nectar, orange (A03BG), fortification agent = Calcium (F09.A0EXH), qualitative info. = Sugar free (F10.A077L), production method = Organic production (F21.A07SE) [2]. Using FoodEx2 is not difficult, but the coding is a process that is always based on a choice by a person and needs to be manually performed.

There are two important rules when using FoodEx2. The first is to have prior knowledge of the system and the second is to choose the right starting point or what type of food it is with regard to FoodEx2 classification, i.e., is it a raw, a derivative or a composite food. This is important because different food categories may have different facet descriptors. By using FoodEx2 to classify and describe foods, it makes it easier to compare data from different data sources and to perform more detailed data analysis [4,5]. Unfortunately, many food composition data and food consumption data, which need to be connected, are lacking since the process of classifying and describing food is performed manually, which is time-consuming and requires a good knowledge of the system. In 2016, European Food Information Resource, EuroFIR, [6] organized a workshop for food matching at the EuroFIR Food Forum 2016, where different European countries presented their results and experience of matching food items. Most countries used FoodEx2 codes, and all of the proposed methods were based on manually searching FoodEx2 data, which is a time-consuming task. Among those that did not use FoodEx2 were a group from Swiss Federal Institute of Technology in Zurich, ETH Zurich, which presented a semi-automatic way of matching food items using food names and text similarity measures applied at the character level, and our group, which presented a promising method for matching food items to food composition data, although it has not been developed to match food items using FoodEx2.

In this study, we introduce a semi-automatic system, called StandFood, to standardize foods according to FoodEx2. The system consists of three parts. The first identifies what type of food is being analyzed (r, d, s, or c). This is the classification part that involves a machine learning (ML) approach [7,8]. The second describes the food using natural language processing (NLP) [9,10] combined with probability theory, which results with the list term or FoodEx2 code for the food. The third combines the result from the first and the second part by defining post-processing rules in order to improve the result for the classification part.

## 2. Materials and Methods 

### 2.1. FoodEx2 Data

From the available FoodEx2 data [2], 5416 instances of food items were selected. Instances are food items that have the attribute “Statef” with the value r, d, s, or c. This attribute indicates the level of the food category represented by the term in the food chain, e.g., a raw, a derivative, a simple composite, or an aggregated composite food. These were selected because we need to determine to which food category a food item belongs before describing a food item. The same instances are then used for the description part.

### 2.2. StandFood

StandFood is a semi-automatic system for classifying and describing foods according to FoodEx2. It consists of three parts. The first one classifies foods into four FoodEx2 categories (groups), two for single foods: raw (r), derivatives (d), and two for composite foods, simple (s) and aggregated (c). For this purpose, it uses a ML approach. The second part is used for describing foods using the FoodEx2 facets, by using a NLP approach combined with probability theory. The third combines the result from the first and the second part by defining post-processing rules in order to improve the result for the classification part.

An evaluation of the StandFood system was made using a dataset from Slovenia of already classified and described foods using FoodEx2 codes. In the dataset, each food item is represented by a food name and a FoodEx2 code, which is manually added by a human expert. StandFood was then used, first to provide the food category to which the item belongs, and second to describe it using FoodEx2 code. This was then compared with the food category and the code that was added manually.

#### 2.2.1. Classification Part

Classification is a supervised ML approach that trains a model using a labeled training set to predict a category (class) from input features [8]. The training set consists of instances (observations) described by features whose category membership is known. An algorithm used for classification is called a classifier and it maps input data to a category. The main goal of such an algorithm is to analyze new unseen instances that are not present in the training set, or to predict their categories. Because the ML supervised algorithms perform well for instances from the training set, their evaluation needs to be performed using a test set that consists of instances not found in the training set. For this purpose, the training set is often randomly split into three portions, the training set, the validation set, and the test set. The training set is used to train a ML supervised algorithm, the validation set is used to select the best performing algorithm (or parameters of the algorithm), and the test set is used to evaluate the performance. Some literature suggests that a typical ratio to split into training, validation, and test sets needs to be made using the 60%/20%/20% or 70%/10%/20% rule [8]. Another approach for evaluating the performance of ML supervised algorithms is to use *k*-fold cross-validation [8]. In this case, the training set is divided into *k* subsets. Each time, one of the *k* subsets is used as the test set and the other *k* − 1 subsets are combined to form a training set. Then the average error across all *k* trials is computed. The advantage of this method is that it is not important how the data is divided. Every data instance gets to be in a test set exactly once, and gets to be in a training set *k* − 1 times. The variance of the resulting estimate is reduced as *k* increases. The disadvantage of this method is that the training algorithm has to start from scratch *k* times, which means it takes *k* times as much computation to make an evaluation. In the case of *k*-fold cross validation, the test sets are validation sets that are used to select the best algorithm (or best parameters of the algorithm). Then, the selected model needs to be evaluated on a test set that consists of new unseen instances. When working with ML supervised algorithms, ensemble learning is used [11], which is the process by which multiple classifiers are combined to solve a particular problem in order to improve on the performance that can be achieved when each of the combined classifiers are used alone.

The StandFood classification part consists of the following three steps:

- Pre-processing of the instances (food items names)
- Feature selection (building a document-term matrix and adding more relevant features)
- Model training

In the case of StandFood, the training set consists of 5416 instances. Each instance is represented by a food item name and the category to which it belongs (r, d, s, or c). In this case, it involves working with text data, which is unstructured and depends on how people express themselves. Before training a model for classification, the data needs to be pre-processed. First, each food item name is pre-processed by changing all letters to lower case, removing punctuations and numbers. Then, for each food item name, tokenization is applied, which is a process of breaking text into words that are called tokens. Each token consists of a string of characters without a space. For example, one instance is “dried vine fruits (raisins etc.)” and its food category is d. After pre-processing, this instance is transformed into “dried vine fruits raisins etc.”. Then, tokenization is applied, and the tokens are: dried, vine, fruits, raisins, and etc. These tokens can then be analyzed by applying lemmatization [12] or stemming [13,14]. From linguistics, lemmatization is the process of grouping together different inflected forms of a word so they can be analyzed as a single item. The uninflected form is called the lemma. In computational linguistics, lemmatization is a process of determining the lemma of a given token (word). It usually works by removing the suffixes of the token in order to return the lemma or dictionary form of the token. Stemming is another approach similar to lemmatization. It works by removing the suffixes of the token in order to give a good approximation to the lemma. For example, the lemma for “dried” is “dry”, while the stem is “dri”. In the case of StandFood, stemming is applied to each token. After pre-processing, each of the instances represents a document that belongs in a text corpus.

**Figure 1:**
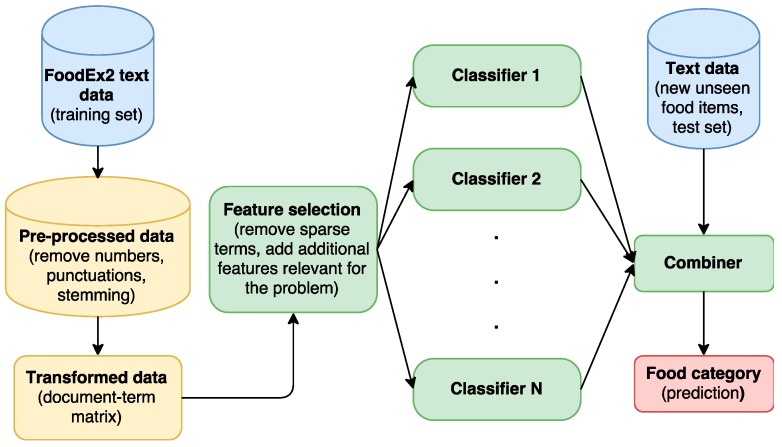
The StandFood classification part flowchart. FoodEx2 names are used as training instances. They are pre-processed by removing numbers and punctuations and stemming. Then, the document-term matrix is built, so it can be used for feature selection. Additional features to the problem are added to the selected features. Different classifiers and ensemble learning are used to obtain a model that can be further used to predict the food category of new unseen food items.

The corpus is then transformed into a document-term matrix. A document-term matrix is a mathematical matrix that describes the frequency of terms that occur in a collection of documents. The matrix gives the relationship between terms and documents, where each row stands for a document (food item name) and each column for a term, and an entry is the number of occurrences of the term in the document. For example, the food item “dried vine fruits (raisins etc.)” is a document, which is a row in the document-term matrix. This document consists of five terms, “dri”, “vine”, “fruit”, “raisin”, and “etc.”, which are the stems of the tokens from the food item name. So, there is an entry 1 on the positions where the raw is “dried vine fruits (raisins etc.)” and the columns are “dri”, “vine”, “fruit”, “raisin”, and “etc.”, because each of these terms are presented exactly once in the food item name. As in most cases when working with text data, the matrix is very sparse; in our case the sparsity is equal to 99%. For StandFood, only the non-sparse entries are used, which means that the terms that only appear in at most 1% of the documents are removed. The document-term matrix consists of 5416 documents, each of which is represented by 276 terms (features). Because the performance of the ML algorithm depends also on the features used to represent the instance, a feature selection is often applied, which is a process of selecting a subset of relevant features to be used to construct a model [8]. In the case of StandFood, removing the sparse terms is a kind of feature selection. However, to improve the model further, four additional features were added. The first is the number of nouns found in the food item name. This is relevant because nouns carry the most information, so more nouns means a higher probability that the food item is a composite food. The second and the third are the number of adjectives and verbs, respectively. Adjectives explain food items in the most specific form (e.g., frozen, fresh) and the verbs are generally related with the method of preparation (e.g., cooked, drained). This is also relevant because if food items consist of adjectives and verbs, there is a higher probability that it is a derivative or composite food since cooking changes the nature of the food. The last feature is the length of the description. A long description usually means a higher probability that the food item is a derivative or composite food. After representing each instance of the training set by the selected features, the next step is to train the model. For this purpose, different classification algorithms can be used. In the case of StandFood, ensemble learning is applied, which combines four classification algorithms: Support Vector Machine (SVM) [15], Random Forest (RF) [16], Boosting (Boosting) [17], and Max Entropy (Maxent) [18]. Using an ensemble of these four algorithms, the model is trained and can be used to predict the category of new unseen instances. A flowchart of the StandFood classification part is presented in Figure 1.

#### 2.2.2. Description Part

After clarifying the category of the food item, it is then necessary to describe it using the FoodEx2 code. The StandFood description part uses a NLP approach combined with probability theory in order to match a food item using its name to a food item that already exists in the FoodEx2. A similar approach was used to match internet recipe ingredients with food composition databases in [19]. However, in the case of StandFood, this approach has been extended.

In StandFood, each food item is described by its name. To describe it using FoodEx2 code, Part-Of-Speech (POS) tagging [20,21,22,23], also called grammatical tagging, is used to identify nouns, adjectives, and verbs. POS tagging is a process of assigning morphological tags or categories (classes) to each token (e.g., NN (noun, singular or mass), VB (verb, base form), VBD (verb, past tense), JJ (adjective), etc.). On each extracted set, lemmatization is applied. The FoodEx2 food names are then searched for all extracted nouns from the POS tagging and each name that consists of at least one of the provided nouns is returned. The result is a subset of food item names from the FoodEx2 data. Then, for each food item name in the subset, POS tagging is used to extract the nouns, adjectives, and verbs. For each set, lemmatization is applied to obtain the lemmas.

For example, if the food item, dried vine fruits (currants, raisins and sultanas), needs to be described with a FoodEx2 code, first POS tagging is applied to extract the nouns, adjectives, and verbs. These sets are then processed by applying lemmatization. The resultant sets are the noun set, which is “vine, fruit, currant, raisin, sultana”, the adjective set, which is empty for this food item, and the verb set, which is “dry”. The 5416 FoodEx2 food names are searched for all extracted nouns, which are in the noun set, in this case for the five nouns, and the food names that consist of at least one of the five extracted nouns are returned. For this food item, 225 FoodEx2 food names are returned. Some of them are: “fruit soup dry”, “African oil palm fruits”, “native currant”, “dried vine fruits (raisins etc.)”, “Malaga raisins flavor”, “juice red currant”, “sultanas flavor”, etc. Each one of the returned FoodEx2 food names is processed by applying POS tagging and lemmatization. If the food item is “mushroom soup”, after POS tagging and lemmatization, the extracted noun set is “mushroom, soup”, while the adjective and the verb sets are empty. The 5416 FoodEx2 food names are searched for the two nouns, and the food names that consist of at least one of the two extracted nouns are returned. For this food item, 65 FoodEx2 food names are returned. Some of them are: “field mushroom”, “canned mushroom”, “mushroom flavor”, “mushroom soup”, “mushroom salad”, “pizza and similar with cheese and mushrooms”, “honey mushrooms”, etc. Each one of the returned FoodEx2 food names is processed by applying POS tagging and lemmatization.

The next step is to define the similarities between the food item name and each of the FoodEx2 food item names that belong to the subset. To do this, an event of similarity between the food item name and each of the returned food item names is defined. Finally, the weight that is assigned to each matching pair is the probability of the similarity event.

For the similarity event, let D_1_ be the food item name we want to describe according to FoodEx2 and D_2_ is a FoodEx2 food item name that belongs to the returned subset. Let us define

*N_i_ = {nouns extracted from D_i_},* *A_i_* = {*adjectives extracted from D_i_*}, *V_i_* = {*verbs extracted from D_i_*}, (1)

where *i* = 1,2.

To find the similarity between these two food items names, an event is defined as a product of two other events

*X* = *N* (*A + V*) (2)

where *N* is the similarity between the nouns found in *N*_1_ and *N*_2_, and *A* + *V* is the similarity between the two sets of adjectives and verbs handled together as *A*_1_ + *V*_1_ and *A*_2_ + *V*_2_. The adjectives and verbs are handled together to avoid different forms with the same meaning. For example, if adjectives and verbs are handled separately, the match “apple dry” and “dried apples” will not be a perfect match. However, for the computer “dry” and “dried” are completely different words that have the same meaning. The same is also true for the singular and the plural form of “apple”. Also, if adjectives and verbs are handled separately, the match “browned bread” and “brown bread” will not be a perfect match because “browned” is a verb and “brown” is an adjective, but both have the same meaning. To avoid this, lemmatization is applied for each extracted noun, verb and adjective, and the similarity event uses their lemmas.

Because these two events are independent, the probability of the event X can be calculated as

*P*(*X*) = *P*(*N*) *P*(*A + V*) (3)

For this, the probabilities of each of the two events need to be defined. Because the problem looks for the similarity between the two sets, it is logical to use the Jaccard index, J, which is used in statistics for comparing similarity and diversity of sample sets [24]. For the similarity between the nouns, the Jaccard index is used, while for the similarity between the adjectives and verbs the Jaccard index is used in combination with Laplace probability estimate [25]; this is because, in some food item names, the additional information provided by the adjectives or verbs can be missed, but the relevant match can be found, so there will be no zero probabilities. The probabilities are calculated as

(4) $$\begin{array}{c} P\left(N\right)=\frac{\left|N_{1}⋂N_{2}\right|}{\left|N_{1}⋃N_{2}\right|}\\ P\left(A+V\right)=\frac{|(A_{1}⋃V_{1})⋂(A_{2}⋃V_{2})|+1}{|(A_{1}⋃V_{1})⋃(A_{2}⋃V_{2})|+2} \end{array}$$

where ⋂ represents the intersection between two sets, which is a set that contains elements from the first set that also belong to the second set (or equivalently, all elements form the second set that also belong to the first set), ⋃ represents the union between two sets, which is the set of all elements from both sets, and |.| is the cardinality of a set, which is a measure of the number of elements in the set. By substituting Equation (4) into Equation (3), we obtain a weight for each matching pair. Finally, the pair with the highest weight is the most relevant found match.

**Figure 2:**
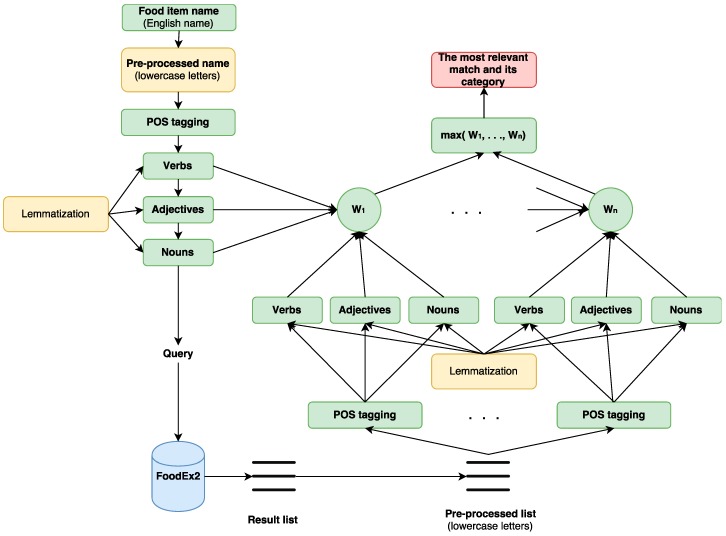
StandFood description part flowchart. For each food item that needs to be described according to FoodEx2, its English name is used. The name is pre-processed by converting it to lowercase letters. Part-Of-Speech (POS) tagging is used to extract its nouns, adjectives, and verbs. The extracted sets are further transformed using lemmatization. Using the extracted nouns, the FoodEx2 data is searched for the names that consist of at least one of the extracted nouns. Then, the resulting list (subset) is pre-processed by converting each food item name to lowercase letters, applying POS tagging to extract the nouns, adjectives, and verbs, and using lemmatization for the extracted sets. Then, the food item that needs to be described according to FoodEx2 is matched with each food item in the resulting list and a weight, W_i_, *i* = 1,.., *n*, which is the probability obtained using Equation (3), is assigned on each matching pair. Finally, the pair with the highest weight is the most relevant one, so it is returned together with its food category from FoodEx2.

In the example of “dried vine fruits (currants, raisins and sultanas)”, the probability of similarity between the food item and each one of the 225 returned FoodEx2 food names must be defined using Equation (3). Finally, the matching pair with the highest weight is the most relevant one. In this example, the most relevant match is “dried vine fruits (raisins etc.)” and its probability weight is 0.4. In the example of “mushroom soup”, the probability of similarity between the food item and each one of the 65 returned FoodEx2 food names must be defined. The matching pair with the highest probability is “mushroom soup”, which is a perfect match, and its probability is 0.5. The flowchart of the StandFood description part is presented in Figure 2.

#### 2.2.3. Post-Processing Rules to Improve the Accuracy of the Classification Part

Results of the description part are used to improve the performance of the classification part. Because for each food item the most relevant item from FoodEx2 is returned, the category of the returned food item can be used for post-processing rules. To do this, StandFood applies the following four rules:

- In the first rule, processes that change the nature of a food product are used. Their definitions are in the technical report of FoodEx2 and include, for example, canning, smoking, frying, and baking. Then, for each process in the list of processes, lemmatization is applied to avoid different word forms of food items names. So, if a food item is classified as raw (r) using the StandFood classification model but its set of adjectives and verbs (their lemmas) consists of at least one cooking process that change its nature, it is automatically changed to a derivative (d).
- In the second rule, if a food item is classified, as being either raw (r) or a derivative (d) and the result from the description part of StandFood is that the most relevant food item is a composite food (s) or (c), it is automatically changed to a composite food.
- In the third rule, if a food item is classified as a simple composite food (s) and the result from the description part of the StandFood system is that the most relevant food item is an aggregated composite food (c), it is automatically changed to an aggregated composite food (c).
- The fourth rule works in reverse, by changing an aggregated composite food (c) to a simple composite food (s).

**Figure 3:**
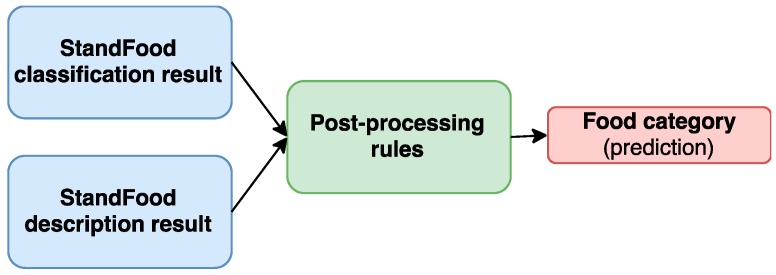
StandFood post-processing part. The results from the StandFood classification and description parts are combined together to improve the accuracy of the classification part for the food category prediction.

The flowchart showing the StandFood post-processing part is presented in Figure 3.

## 3. Results

For the classification part of StandFood, the RTextTools package for R programming language was used. More details about how to use this package are available in RTextTools: A supervised learning package for text classification [26]. For the description part and for extracting information about additional features used in the classification, a coreNLP package for R programming language was used [27].

### 3.1. StandFood Classification Results

The training set of 5416 instances is described using 280 features, from which the first 276 are the non-sparse terms selected from the document-term matrix and the last four are the additional features, which are the number of nouns, adjectives, and verbs, and the length of the food item’s name. The problem remains how to classify instances into four categories (r, d, s, and c), which makes it a multiclass classification problem. The distribution of the categories in the training set is: r (2558), d (1795), c (309), and s (754). To this data, different classification algorithms are applied. To evaluate the algorithmic performance, it is possible to analyze performance metrics such as precision, recall, and accuracy. For classification tasks, the terms true positives (TP), true negatives (TN), false positives (FP), and false negatives (FN), were used to compare the results of the classifier with results provided by a human expert. The terms positive and negative refer to the classifier prediction, while the terms true and false refer to the prediction made by the human expert. Using these terms the precision, recall, and accuracy of the classifier are defined as

(5) $$Precision=\frac{TP}{TP+FP}\;Recall=\frac{TP}{TP+FN}\;Accuracy=\frac{TP+TN}{TP+TN+FP+FN}$$

Precision for a category is the number of true positives (the number of items correctly labeled as belonging to the category) divided by the total number of items labeled as belonging to the category (the sum of true positives and false positives, which are items incorrectly labeled as belonging to the category). Recall is defined as the number of true positives divided by the total number of items that actually belong to the category (the sum of true positives and false negatives, which are items which were not labeled as belonging to the category but should have been). In the classification task, a precision score of 1.00 for a category C means that every item labeled as belonging to category C does indeed belong to category C (but says nothing about the number of items from category C that were not labeled correctly), whereas a recall of 1.00 means that every item from category C was labeled as belonging to category C (but says nothing about how many other items were incorrectly also labeled as belonging to category C). Usually, precision and recall are not discussed separately. Accuracy is the ratio of the corrected predictions and the total number of predictions made. 

In the case of StandFood, different classification algorithms were applied, and the obtained models were evaluated using a 10-fold cross validation. The algorithms used were: support vector machine (SVM) [15], scaled linear discriminant analysis (SLDA) [28], random forest (RF) [16], maximum entropy (Maxent) [18], boosting (Boosting) [17], bagging (Bagging) [29], classification tree (TREE) [30], and neural networks (NNET) [31]. Different classification algorithms were used to select the best classification model. The selected algorithms are the most commonly used algorithms for the classification task described in the ML literature and the accuracy of each of the models using a 10-fold cross validation is presented in Table 1.

From the results, it becomes clear that SVM, RF, Maxent, Boosting, and Bagging outperform the other algorithms, because from a practical point of view they have better accuracy. To enhance the accuracy even further, ensemble learning was used. Ensemble learning refers to whether multiple algorithms make the same prediction concerning the category of an instance. For the StandFood classification part, ensemble of the four best performing algorithms: SVM, RF, Maxent, and Boosting, using a majority vote strategy, was used [32]. The majority vote strategy uses the category provided by each algorithm (classifier) and then chooses the category that receives the largest total vote. The obtained accuracy of the ensemble using a 10-fold cross validation is 91.20%. Also, different ensembles combining three or five of the best performing algorithms were used, but the results are not practically significantly different from the ensemble that combines the four best performing algorithms. In the context of scientific research, it is instructive to make a distinction between statistical significance and practical significance. For example, the obtained accuracy of the ensemble of the five best performing algorithms using a 10-fold cross validation is 90.50%. If the results for both ensembles, using the four or five best performing algorithms, respectively, are compared, between them statistical significance can be found, but in a practical sense, this difference is not significant. Because of this, StandFood uses an ensemble that combines the four best performing algorithms. The evaluation of the trained model using a 10-fold cross validation gave the most promising results and the model can be used to classify new unseen instances. To evaluate StandFood properly, 532 new instances from the food composition database were used. The results for precision and recall are presented for each category separately in Table 2.

The recall for the raw category (r) is 0.99, which means that 99% of the raw instances in the test set are assigned to the raw category. The precision for the raw category (r) is 0.72, from which it follows that there are many false instances assigned to this category. For the derivative category (d), the precision and recall are 0.81 and 0.81, respectively. For the aggregated composite food category (c), the precision is 0.75, which indicates that there are false instances also assigned to this category. Analyzing the false instances that appear in this case showed that these instances belong to the simple food category (s) and the StandFood classification part assigned them as aggregated composite food (c). The recall for the aggregated food category (c) is 0.67, which indicates that only 67% of the aggregated composite food instances presented in the test set are assigned to that category. Looking at the results, the majority of improperly classified instances from the category are assigned to the raw category (r), which influences the precision of the raw category (r). The precision of the simple composite food category (s) is 0.95, which means that there are only a few false instances assigned to that category, while the recall is 0.57, which indicates that only 57% of the simple composite food instances from the test set are assigned to that category. In most cases, the improperly classified instances from that category are assigned to the raw (r) or derivative (d) categories.

Table 3 gives the results from the StandFood classification part of eight randomly selected but correctly classified instances, two per food category.

### 3.2. StandFood Description Results

In the description part, the main goal is to describe food items according to FoodEx2 and to find the most relevant food item that exists in the FoodEx2 data. As well as the FoodEx2 code, the food category (r, d, s, and c) is returned, which is further combined with the result from the classification part to improve its performance.

The StandFood description part is an NLP approach combined with a probability theory. For its evaluation, for each food item from the test set, it returns the most relevant food item from FoodEx2. Then, the obtained FoodEx2 code is compared with the FoodEx2 code that was manually assigned to the food item by a human expert. Evaluating the description part on the test set, 79% of the instances obtain relevant FoodEx2 codes. In the set of instances that obtain a relevant FoodEx2 code, there are instances for which the FoodEx2 code assigned by StandFood is the same as the FoodEx2 code provided by a human expert. There are also instances in which FoodEx2 codes are different but are close together in the FoodEx2 hierarchy, so they are still related to the same food. For example, for the food item “mutton/lamb meat (ovis aries)”, the FoodEx2 code assigned by StandFood is A01RK, which is related to the food item “Lamb fresh meat”, while the FoodEx2 code provided by the human expert is A01RH, which is related to the food item “Sheep muscle”. In this case, both codes are very close in the FoodEx2 hierarchy, and A01RH is a parent for A01RK. There are instances for which the manually assigned code is incorrect, while the StandFood system returns the correct FoodEx2 code; that can happen because a human expert is not familiar enough with the details in FoodEx2. In addition, StandFood can return more than one relevant choice, when more food items have the same highest weight. In such cases, the user should select which of them is the most relevant one. As an example, ten randomly selected instances are presented in detail in Table 4.

For the first seven food items, the FoodEx2 code obtained by StandFood is the same as the FoodEx2 code manually assigned. Having the true FoodEx2 code, more information for a food item can be obtained by the FoodEx2 data with a simple search using the FoodEx2 code. For example, “mushroom soup” is described as A041R#F02.A06GY$F04.A0ETG$F28.A07MR$F28.A0BA1. This means that the product is “mushroom soup”, with the part-nature descriptor (F02) “soups ready-to-eat (as part-nature)” (A06GY), then it has an ingredient descriptor (F04) set to “fungi” (A0ETG) and two process descriptors (F28), one for “reconstitution from concentrate, powder or other dehydrated form” (A07MR) and the other for “cooking and similar thermal preparation processes” (A0BA1). For the eighth food item, the FoodEx2 code obtained by StandFood and manual code differs. For StandFood, it is a perfect match, whereas for manually assigned FoodEx2 code, food item is “wheat semolina” (A004F). The two codes are close in the FoodEx2 hierarchy. However, the StandFood code is more relevant since the code is for “wheat flour durum” that exists in FoodEx2. For the ninth food item, StandFood again returns a perfect match. The manually assigned FoodEx2 code for this food item is “gingerbread dough” (A009Q) and has a final-preparation descriptor (F14) set to “baking” (A07GX). However, “gingerbread” already exists in FoodEx2 data and there is no need to recode it (FoodEx2 recommends to code new food items only if they do not exist in the FoodEx2 data). Also, if there is a need to code new food items, the FoodEx2 recommends not to use facet descriptors from F13 to F16 (F13—cooking method, F14—final preparation, F15—preservation technique, F16—structural treatment), and instead use only F28 i.e., the process descriptor. In this case, the code given by StandFood is more relevant than the manually assigned code. In the last example, the manually assigned FoodEx2 code is one of the codes returned by StandFood. In this case, there are several food items from the FoodEx2 data that have the same weight, which is the highest weight obtained by the equation used in the description part, so they are all returned as relevant matches. The user should select which of the returned matches is the most relevant one. In the case of StandFood, 10% of the food items that received a relevant FoodEx2 code have a more relevant code than the code assigned manually; that can happen because a human expert is not familiar with the details in FoodEx2.

The idea used in the description part of StandFood is based on NLP, or using text similarity measures. StandFood uses short segments of texts, so it can not use standard text similarity measures [33] because they fail when tasked with computing the similarity between two very short segments of text [34]. In this work, different similarity measures, including the Jaccard index applied on the character level between the names of two food items, Jaccard index applied on the word level, negative Kullback-Leibler (KL) divergence [35] were used, but none gave promising results. The problem of using short segments of text is explained in [34]. The StandFood description part gives good results because domain modeling is applied, and the model combines text similarity measures using probability theory.

### 3.3. StandFood Post-Processing Rules

After obtaining the result from the StandFood description part, the food category (r, d, s, or c) of the most relevant match is further combined with the obtained food category from the StandFood classification part. By applying the four post-processing rules defined in Section 2.2.3, the newly obtained precision and recall for each food category using the test set are obtained in Table 5.

A comparison between Table 2 and Table 5 reveals improvements in the precision and recall of each category and 89.28% of the instances from the test set are correctly classified. As an example, post-processing of seven randomly selected foods were carried out (Table 6).

## 4. Discussion

The main benefit of using StandFood is that missing FoodEx2 data in a lot of food composition data sets can be obtained and being a semi-automatic system, it significantly reduces the time needed to code food items. When the FoodEx2 data is available, it is easier to compare the food data presented in the data set with food data from another study, or combine them. StandFood gives 79% correctly classified and described instances, which means that it is a promising tool that can be used. However, 21% of the instances are not correctly described (in them there are instances that are correctly classified), and this happens according to the facts that food items do not exist in FoodEx2, food items are typical for some cultures, or food description is not complete and detailed as possible. StandFood is a semi-automatic system, which means that the user needs to check the result that is obtained. Also, it is seen that there are some cases when the StandFood system gives more a relevant FoodEx2 code than a human expert. This happens because human experts manually code food items with facet descriptors to describe them in detail, when food items already exist in FoodEx2, and they are not familiar with the details in FoodEx2, which makes this an ongoing problem, as FoodEx2 will evolve with new entries all the time. When a new food item is not present in FoodEx2 it needs to be coded (in most cases they are composite foods), which will be our future work. In some cases, StandFood returns the ingredients of the foods that are presented in the food description that further need to be coded with some other facet descriptors. For example, for the food item “baked potatoes with parsley”, it returns “potatoes” and “parsley”. To code the new food items (composite foods), it is better to find recipes for them and try to extract all useful information that can help the process of coding, starting from the used ingredients, cooking processes, etc. For our future work, it is planned to code these cases following the recommendations given in the FoodEx2 technical report.

Using the trained model on the FoodEx2 data, there are some weaknesses when it is applied on new unseen instances. The main reason why this happens is because of the intrinsic ambiguity of language, meaning that different people may interpret the name of a food item differently. For example, the instance “potato boiled” is a derivative food; however, the StandFood system assigns it to the raw category. This happens because “boiled” is not used as a feature for classification and it is one of the sparse terms that are removed because it appears only in 3 out of the 5416 instances. Using synonyms is also problematic (e.g., mushrooms tinned (r), mushrooms canned (d)), and this happens because “canned” exists as a feature extracted from the FoodEx2 data. Despite the weakness of the trained model, it can still be used because the StandFood classification part includes post-processing rules in order to improve its performance.

According to the time needed to code the new 532 food items, in the case of StandFood minutes (five minutes) are required because the current version of StandFood is programmed in a sequential way. Parallel programming would allow the same task to be carried in seconds. A human expert takes on average ten minutes per item, or 5320 min for 532 food items, or 11 days if a human expert works eight hours per day. However, the result from StandFood needs to be checked and in the case of several options to choose the correct one. For 532 food items, thorough checking and choosing the correct result when several options were given took one working day (eight hours).

StandFood depends on the food item name. Since different people may interpret the name of a food item in a slightly different way, a better explanation in the food item name would provide a higher probability of finding the correct FoodEx2 code. At present, the system works by using the English food item name. In our case, the names of food items were translated from Slovenian by a non-native English speaker, which also represents a potential weakness because the description might include errors or strange translations. In future work, it is also planned to upgrade StandFood so it can be used with different languages, by automatically translating food item names into English using the Google Translation Application Program Interface, API. The Google API is not perfect, especially when local food item names can be very peculiar and difficult to translate. Translation will work better when the food item name is as complete and detailed as possible, so we will try to find a way to obtain more detailed and complete food item names that will be used in translation and then by StandFood. Also, we will try to link other food classification systems (i.e., LanguaL or “language of food”, an international framework for food description) to FoodEx2 in order to improve the result of StandFood.

In practice, StandFood can be used to find missing FoodEx2 codes for food composition data and food consumption data that are basic resources that need to be linked and combined for dietary assessment methods. For the near future, we are planning to implement StandFood as a web service, which will be freely available for the wider research community.

## 5. Conclusions

This paper presents a semi-automatic system for classifying and describing food items according to the FoodEx2 standard, called StandFood. It consists of three parts. The first classifies food items into one of four FoodEx2 food categories: raw, derivative, simple composite food, and aggregated composite food. It uses a machine learning approach to train a model for classification. The second step describes food items using FoodEx2 codes. It uses a natural language processing approach based on text similarity measures combined with probability theory. The third combines the result from the first and the second part by defining post-processing rules in order to improve the result for the classification part. Evaluation results of the StandFood shows how the system gives promising results and can be used to classify and describe food items according to FoodEx2. Using StandFood, it is possible to find missing FoodEx2 codes in food composition databases and food consumption data, which allows the users to compare and combine them.

## Figures and Tables

**Table 1:** Classification accuracy for each machine learning (ML) algorithm using 10-fold cross validation. SVM—support vector machine, SLDA—scaled linear discriminant analysis, RF—random forest, TREE—classification tree, NNET—neural networks.

Metric	SVM	SLDA	RF	Maxent	Boosting	Bagging	TREE	NNET
Accuracy (%)	88.50	72.41	88.95	89.21	85.88	83.47	69.02	77.12

**Table 2:** Precision and recall for each food category using the evaluation set.

Category	Precision	Recall
r	0.72	0.99
d	0.81	0.81
c	0.75	0.67
s	0.95	0.57

**Table 3:** Correctly classified instances by the StandFood classification part.

Food Item	Category
Barley grains	r
Mandarins (*Citrus reticulata*)	r
Buckwheat flour	d
Oat flakes	d
Fruit compote	s
Marmalade, mixed fruit	s
Rice and vegetables meal	c
Mushroom soup	c

**Table 4:** Results from the StandFood description part for ten randomly selected instances. Food item represents the name of the food item in the test set. StandFood relevant FoodEx2 item is the name of the most relevant match that exists in the FoodEx2 found by the StandFood. StandFood FoodEx2 code is the FoodEx2 code of the most relevant match found by the StandFood. Manual FoodEx2 code is the FoodEx2 code that was manually assigned to that food item by a human expert.

Food Item	StandFood FoodEx2 Code	StandFood Relevant FoodEx2 Item	Manual FoodEx2 Code
Mushroom soup	A041R	Mushroom soup	A041R
Prepared green salad	A042C	Mixed green salad	A042C
Meat burger	A03XF	Meat burger no sandwich	A03XF
Yeast	A049A	Baking yeast	A049A
Brown sauce (gravy, lyonnais sauce)	A043Z	Continental European brown cooked sauce gravy	A043Z
Cow milk, <1% fat (skimmed milk)	A02MA	Cow milk skimmed low fat	A02MA
Supplements containing special fatty acids (e.g., omega-3, essential fatty acids)	A03SX	Formulations containing special fatty acids (e.g., omega-3 essential fatty acids)	A03SX
Durum wheat flour (semola)	A004C	Wheat flour durum	A004F
Gingerbread	A00CT	Gingerbread	A009Q$F14.A07GX
Cherry, fresh	A01GG	Cherries and similar	A01GK
	A01GH	Sour cherries	
	A01GK	Cherries sweet	
	A0DVN	Nanking cherries	
	A0DVP	Cornelian cherries	
	A0DVR	Black cherries	

**Table 5:** Precision and recall for each food category after applying the post-processing rules.

Category	Precision	Recall
r	0.85	0.99
d	0.90	0.84
c	0.82	0.87
s	0.97	0.83

**Table 6:** Food categories for seven food items after post-processing rules. Classification part category is the food category assigned by the StandFood classification part. Post-processing category is the category assigned after post-processing rules.

Food Item	Classification Part Category	Post-processing Category
Cabbage Chinese boiled	r	d
Marzipan	r	s
Gingerbread	r	c
Water, bottled, flavored, citrus	d	s
Salad, tuna-vegetable, canned	d	c
Multigrain rolls	c	s
Croissant, filled with jam	s	c

## Abbreviations

The following abbreviations are used in this manuscript:

EFSA	European Food safety Authority
FN	False negatives
FoodEx1	Food classification and description system for exposure assessment (version 1)
FoodEx2	Food classification and description system for exposure assessment (version 2)
FP	False positives
Maxent	Maximum Entropy
ML	Machine Learning
NLP	Natural Language Processing
NNET	Neural Networks
RF	Random Forest
SLDA	Scaled Linear Discriminant Analysis
StandFood	Food Standardization System
SVM	Support Vector Machine
TN	True negatives
TP	True positives
TREE	Classification tree
